# No Association Between Autonomic Functioning and Psychopathy and Aggression in Multi-Problem Young Adults

**DOI:** 10.3389/fpsyg.2021.645089

**Published:** 2021-03-16

**Authors:** Josjan Zijlmans, Reshmi Marhe, Laura van Duin, Marie-Jolette A. Luijks, Floor Bevaart, Arne Popma

**Affiliations:** ^1^Department of Child and Adolescent Psychiatry, Amsterdam University Medical Center, Amsterdam, Netherlands; ^2^School of Social and Behavioural Sciences, Erasmus University Rotterdam, Rotterdam, Netherlands

**Keywords:** aggression, antisocial, psychophysiology, young adulthood, psychopathy

## Abstract

**Background:**

Aberrant functioning of the autonomic nervous system (ANS) is an important factor in the occurrence of antisocial behavior. Baseline autonomic functioning and the responsivity of the ANS have been related to psychopathic traits and aggression. Here we investigated whether a naturalistic sample of male multi-problem young adults (age 18–27) present with similar autonomic deficits in relation to their psychopathy and aggression as previous studies observed in clinical samples.

**Methods:**

In a sample of 112 multi-problem young adults, baseline autonomic functioning and autonomic responsivity to emotional stimuli were assessed through four physiological measures: heart rate, respiratory sinus arrhythmia, pre-ejection period, and skin conductance. 27 control participants were included primarily to assess whether the task worked appropriately. Participants watched a neutral 5 min video to assess baseline autonomic functioning and watched two sad clips to assess autonomic reactivity to sadness. We investigated the association between autonomic functioning and self-reported psychopathic traits and aggression within the multi-problem group.

**Results:**

We found no significant associations between autonomic functioning and psychopathy and aggression.

**Conclusion:**

These null-findings highlight the importance of research in naturalistic samples in addition to research in clinical and general populations samples and underscore the complexity of translating research findings into practical and clinical implications.

## Background

The autonomic nervous system (ANS) has long been implicated to play an important role in the occurrence of psychopathy and aggression. It consists of two branches that counteract each other, which combined activities should result in maintaining homeostasis. The sympathetic nervous system (SNS) activates the body in response to stressors and can be measured through heart rate (HR), the pre-ejection period (PEP; time between onset of ventricular depolarization and opening of the aortic valves), and skin conductance level (SCL) (van Lien et al., 2013), where higher HR, lower PEP, and higher SCL represent increased sympathetic activity. The parasympathetic nervous system (PNS) has an inhibitory function on the SNS and thus (re)turns the body to rest. The PNS can be measured through HR and respiratory sinus arrhythmia (RSA; the variability in heart rate between expiration and inspiration) (Grossman and Taylor, 2007), where lower HR and higher RSA represent increased parasympathetic activity. Note that as HR is influenced by both the SNS (HR increase) and PNS (HR decrease) it is not a pure marker of either two. Historically, two dominant theories exist concerning the relationship between ANS activity and reactivity, and antisocial behavior. Low arousal theory proposes that individuals with a hypoactive ANS attempt to elevate their arousal to a preferred level by displaying antisocial behavior. Alternatively, fearlessness theory posits that low arousal is indicative of a lack of fear for negative consequences of actions, resulting in increased engagement in antisocial behavior (Raine, 1993). Both theories predict that resting ANS activity is attenuated in antisocial behavior.

For decades, research has been performed on resting HR, which is the most thoroughly investigated biological correlate of antisocial behavior. Low resting HR has been meta-analytically shown to be related to various antisocial disorders and behaviors including conduct disorder/oppositional defiant disorder (CD/ODD), offending, aggression, and psychopathy (Lorber, 2004; Ortiz and Raine, 2004; Portnoy and Farrington, 2015). However, effect sizes of the relationship have been shrinking with time (Portnoy and Farrington, 2015) and some recent, large-scale studies have failed to find significant associations (Oldenhof et al., 2018; Prätzlich et al., 2018). The relationship between other resting measures of the ANS and antisocial behavior has not been investigated as intensively as HR, but studies suggest that increased resting PEP is related to conduct problems and aggression (Beauchaine et al., 2013); reduced resting SCL is related to psychopathy and conduct problems (Lorber, 2004); and reduced resting RSA is related to externalizing problems and antisocial behavior (Beauchaine et al., 2007; de Wied et al., 2009; Graziano and Derefinko, 2013).

Similarly, diminished reactivity of the ANS to stressors (e.g., movies to elicit emotional reaction or loud noises to elicit a startle response) has been found to be related to antisocial behavior. A meta-analysis showed that HR reactivity during stressors is related to antisocial behavior (Ortiz and Raine, 2004); less PEP reactivity has been observed in conduct disorder (Beauchaine et al., 2007); reduced SCL reactivity has been found to be related to conduct problems and aggression (Herpertz et al., 2001); and reduced RSA reactivity has been demonstrated in conduct problems (Marsh et al., 2008). Autonomic reactivity to negative emotions seems to be most affected (Beauchaine et al., 2019). Possibly this is due to antisocial populations experiencing deficits in the ability to recognize sadness and fear (Marsh and Blair, 2008). In antisocial sample, sadness specifically has been found to be related to decreased autonomic reactivity (Marsh et al., 2008; de Wied et al., 2012). In sum, there is evidence for associations between antisocial behavior and both resting and reactivity measures of the ANS. However, some null-results and discordant findings are present in the literature (e.g., Zahn and Kruesi, 1993; de Wied et al., 2009) and it is unclear whether results hold up across types of antisocial behavior, age ranges, and populations.

As for differences in populations, the greatest focus in research on the relationship between antisocial behavior and autonomic functioning has been on children with CD and ODD, or adolescents with psychopathic traits and aggressive problems. There is a need to further research this relationship in adult samples to (dis)confirm the hypothesis that aberrant autonomic functioning is related to antisocial behavior across the life-course. Thus far, especially little emphasis has been given to the specific period of young adulthood, even though it is now regarded as a distinct developmental stage in which both psychosocial and neurobiological changes occur (Arnett, 2000). Vulnerable populations transitioning into adulthood may be especially relevant to study (Osgood et al., 2010) as they are at an increased risk to persevere in their antisocial behavior or even develop it further (Hill et al., 2018). Multi-problem young adults (18–27) are such a vulnerable population: they lack a stable income, do not have the prerequisites to get a job, have problems in multiple life domains, and 66% have had Child Protection Service () interference, chiefly due to judicial problems before age 18 (van Duin et al., 2017). As they form a naturalistic sample with heterogeneous problems, we can expect their psychopathic traits and aggressive behavior to vary accordingly. This allows for a dimensional approach that includes the full range of psychopathy and aggression and optimizes power.

Thus, in the current study we sought to investigate in a naturalistic sample of multi-problem young adults whether (1) resting measures of the ANS are related to psychopathic traits and aggression, and whether (2) ANS reactivity to emotional stimuli is related to psychopathic traits and aggression. We included a group of community controls primarily to assess whether the task worked appropriately and to exploratively investigate whether multi-problem young adults as a group perform differently compared to a community population. We expected to find lower resting levels of the ANS, as well as lower reactivity of the ANS, to be related to both psychopathic traits and aggression.

## Materials and Methods

### Participants

Participants were 127 male multi-problem young adults [part of a larger study (Luijks et al., 2017)]. Multi-problem young adults are characterized by a lack of income and education, as well as problems in multiple life domains such as addiction, mental health problems, and judicial problems. In our sample, 83% has a criminal record and self-reported delinquency ranges from 10 to 30% in the last 6 months and from 61 to 86% during lifetime, depending on the type of offense (see Table 1). In addition, most participants had Childhood Protective Service interference during their childhood (mainly due to judicial problems) and suffered from multiple adverse childhood experiences. See (Zijlmans et al., 2021) for a more extensive description of the sample. Participants were recruited at the start of day treatment program *De Nieuwe Kans* (DNK; translated as “New Opportunities”). DNK provides a multimodal approach, which aims to increase self-sufficiency and ultimately decrease negative outcomes such as recidivism of multi-problem young adults. DNK is not part of the health care system, but rather is funded by the municipality. It aims to reintegrate participants back into society through education or employment while at the same time reducing recidivism. The program employs cognitive behavioral techniques and rehabilitation components, such as cognitive skills training, drug treatment, and education. Most participants are referred to DNK via a municipal agency (Dutch: *Jongerenloket*) where young adults apply for social welfare. Other participants are referred by youth care, probation services, or social organizations. The goal of DNK is to help young adults reenter education or the job market.

**TABLE 1:** Questionnaire and autonomic baseline measures.

	Multi-problem young adults (*N* = 112)		Healthy controls (*N* = 22)		
		
	*M*	*SD*	*M*	*SD*	*p*
Age (years)	22.42	2.38	22.51	2.45	0.877
IQ	82.64	10.35			
**Education**					
No secondary education	93%		0%		
Secondary education following	0%		42%		
Secondary education finished	7%		58%		
**Psychopathy**					
YPI grandiose manipulative interpersonal	11.39	3.96	13.14	3.50	0.061
YPI affective callous-unemotional	10.65	3.44	11.76	3.27	0.176
YPI impulsive-irresponsible behavioral	12.27	3.27	12.33	3.07	0.938
YPI total	34.32	8.12	37.24	6.46	0.122
**Aggression**					
RPQ Reactive aggression	11.39	4.57	9.23	5.34	0.050
RPQ Proactive aggression	5.44	4.42	4.14	4.54	0.211
RPQ Total	16.83	7.88	13.36	9.43	0.070
Cannabis Use					
Cannabis use past 30 days	13.93	13.51	4.50	7.02	<0.001
Years of regular cannabis use	4.18	3.73	1.30	2.72	<0.001
**Alcohol Use**					
Alcohol use past 30 days	3.23	5.74	4.86	7.98	0.257
Years of regular alcohol use	2.17	3.49	4.50	7.02	0.142
**Self-reported delinquency**					
Destruction/public order offense—lifetime	71%				
Property offense—lifetime	86%				
Aggression/violent offense—lifetime	71%				
Drug offense—lifetime	61%				
**Autonomic resting measures**					
Heart rate (bpm)	65.32	9.21	66.81	9.48	0.491
Respiratory sinus arrhythmia	94.47	42.40	89.50	41.12	0.614
Pre-ejection period (ms)	111.85	16.64	111.74	25.70	0.979
Skin conductance level (μS)	5.03	3.19	3.93	2.49	0.129

*N = number (of participants), M = mean, SD = standard deviation.*

Additionally, 27 age and gender (all participants were male) group matched controls were included in the study. Controls were selected to have average education. Of the multi-problem young adults, 4 were excluded because they failed to complete the task and 11 were excluded because of technical errors with the measuring equipment. Additionally, 5 control subjects were excluded because of technical errors with the measuring equipment. Thus, the final sample included 112 multi-problem young adults (mean age 22.4) and 22 controls (mean age 22.5).

### Instruments

As a measure of psychopathic traits, we employed the Youth Psychopathy Inventory—Short Version (van Baardewijk et al., 2010). The YPI-SV is a self-report measure that distinguishes three factors of psychopathy: an affective callous-unemotional factor, a behavioral impulsive-irresponsible factor, and an interpersonal grandiose-manipulative factor. It has been validated in young adults (Colins and Andershed, 2015). As a measure of aggression, we employed the Reactive Proactive Questionnaire (Raine et al., 2006; Cima et al., 2013). The RPQ consists of two subscales: reactive aggression and proactive aggression. It has been validated in youngsters and adults (Cima et al., 2013). We used the Measurements in the Addictions for Triage and Evaluation Questionnaire (MATE) (Schippers et al., 2010) to assess current and historic cannabis and alcohol use. In order to measure intelligence, we used the short form of the Wechsler Adult Intelligence Scale third version (WAIS-III SF) consisting of four subtests (Blyler et al., 2000): digit symbol coding, information, block design, and arithmetic. We assessed delinquency with the WODC self-reported delinquency questionnaire (Van der Laan and Blom, 2006). The WAIS-III-SF and self-reported delinquency questionnaire were only assessed in the multi-problem group.

### Task and Procedure

Participants were first shown a 5 min excerpt from the video *Coral Sea Dreaming* (Small World Music Inc.) to assess resting levels of the ANS. Previous research has shown that watching this video results in a better measurement of resting levels than sitting quietly (Piferi et al., 2000). Then, participants watched two sad film clips intended to evoke an empathic response to the emotion sadness. One clip involves a boy crying because he did not make the selection of a soccer team (Mohammed) the other clip portrays a boy crying over the loss of his father (Champ). Previous research has shown that both clips reliably evoke sadness in participants (Gross and Levenson, 1995; de Wied et al., 2009), respectively. Before each clip, a 1 min excerpt of the *Coral Seas Dreaming* video was shown to be used as baseline. The clips were counterbalanced across participants. After each clip, participants indicated how they thought the boy in the clip felt by rating the emotions happiness, anger, fear, and sadness on a 5-point Likert scale.

Measurements were performed in the Erasmus Behavioral Lab of the Institute for Psychology at the Erasmus University Rotterdam. Participants were seated in a comfortable chair in a sound-attenuated room with dimmed lights. A trained researcher gave a standardized explanation to each participant concerning the entire protocol. Additionally, a short introduction appeared on the screen before each film clip, indicating when participants simply had to watch the clip (in the case of Coral Sea Dreaming) or also had to answer a few questions about it (in the case of Mohammed and Champ) concerning what emotion they thought the boys in the videos showed and what emotions they themselves experienced while watching the videos.

### Physiological Recording and Processing

We used the VU Ambulatory Monitoring System [VU-AMS; (Klaver et al., 1994)] to record both the electrocardiogram (ECG) and impedance cardiogram (ICG), as well as the level of skin conductance. Five electrodes were placed on the chest, two on the back, and two on the second phalanges of the non-dominant hand according to the VU-AMS manual^1^.

Data processing was performed with the VU-AMS Data, Analysis, and Management Software (VU-DAMS). Heart rate (HR) was assessed by automated counting of the R-peaks; respiratory sinus arrhythmia (RSA) was defined as the longest period between heart beats during expiration minus the shortest period between heart beats during inspiration; pre-ejection period (PEP) was defined as the time between the onset of left ventricular depolarization and opening of the aortic valve; skin conductance level (SCL) is presented in microSiemens.

### Data Analysis

Data analysis was performed in IBM SPSS 21. To assess whether the task validly elicited sadness-related physiological reactions we performed paired-sample *t*-tests comparing physiological activity during baseline and film clips for each physiological measure (HR, RSA, PEP, SCL). We tested whether the responses differed between the clips by performing paired-sample *t*-tests.

For the questionnaire data and physiological data, we used independent sample *t*-tests with group as independent variable to compare the multi-problem group with the community control group. For the analysis of the behavioral responses, we used Mann-Whitney *U*-tests to compare the groups. We used Pearson correlations to establish the relation between psychopathic traits and aggression and established variance inflation factors to assess whether multicollinearity would be an issue for further analyses. We used linear regression analyses to investigate the relation between psychopathic traits, aggression, and physiological measures within the multi-problem group. We performed Bayesian analyses using JASP 0.11 (JASP Team, 2020) to assess whether null results were indicative of evidence in favor of null hypotheses or indicative of a lack of evidence in either direction.

## Results

### Task Validity: Physiological Responses to Sadness Clips

Paired sample *t*-tests showed that there were significant autonomic changes in response to the sadness clips. HR decelerated in both Mohammed (*M* = −1.61, *t* = −3.89, *p* < 0.001) and Champ (*M* = −2.63, *t* = −7.74, *p* < 0.001); RSA lowered in both Mohammed (*M* = −12.15, *t* = −2.86, *p* < 0.01), and Champ (*M* = −15.56, *t* = −5.35, *p* < 0.001); SCL lowered in both Mohammed (*M* = −0.43, *t* = −6.35, *p* < 0.001) and Champ (*M* = −0.27, *t* = −4.63, *p* < 0.001). We found no significant changes in PEP (both ps >0.05). Paired-sample *t*-tests between the difference scores for Mohammed and Champ indicated no significant differences between the autonomic responses to the clips, except for HR. The HR deceleration was larger for Champ than for Mohammed (*M* = −0.99, *t* = −2.16, *p* < 0.05).

### Correlations Between Psychopathic Traits and Aggression and Variance Inflations Factors

Within the multi-problem group, we found significant correlations between most of the psychopathy and aggression subscales (see Table 2 for an overview). Variance inflation factors indicated no issues of multicollinearity (all VIFs <1.8).

**TABLE 2:** Correlations between measures of psychopathy and aggression (multi-problem group only).

	Proactive aggression	Reactive aggression	Total aggression	YPI Interpersonal	YPI Affective	YPI Behavioral
**Proactive aggression**						
Reactive aggression	0.539**					
Total aggression	0.882**	0.873**				
YPI Interpersonal	0.217*	0.359**	0.324**			
YPI Affective	0.133	0.199*	0.187*	0.356**		
YPI Behavioral	0.474**	0.461**	0.528**	0.433**	0.301**	
YPI Total	0.353**	0.445**	0.451**	0.813**	0.719**	0.741**

**Correlation is significant at the 0.01 level (2-tailed).

*Correlation is significant at the 0.05 level (2-tailed).

### Relation Between ANS Functioning and Psychopathic Traits and Aggression in the Multi-Problem Group

Within the multi-problem group, we performed four linear regression analyses with HR, RSA, PEP, and SCL as outcome measures. In each model, the psychopathy and aggression subscales were entered as predictors. None of these models were significant (all *p*s >0.05; see Table 3 for an overview). Bayesian analyses indicate substantial evidence in favor of the null hypotheses with Bayes factors (*BF*_10_; model compared to null model) ranging from 0.06 to 0.10.

**TABLE 3:** ANS baseline regression models within multi-problem young adults (*N* = 112).

Outcome	Predictor	β	*p-value*β	*R*^2^	*p*-value *R*^2^	*BF*_10_
HR	Grandiose manipulative	−0.035	0.751	0.073	0.155	0.101
	Callous-unemotional	−0.011	0.913			
	Impulsive irresponsible	0.243	0.044			
	Reactive aggressive	−0.015	0.899			
	Proactive aggressive	−0.268	0.029			
RSA	Grandiose manipulative	0.117	0.298	0.055	0.307	0.045
	Callous-unemotional	−0.163	0.118			
	Impulsive irresponsible	−0.142	0.241			
	Reactive aggressive	0.178	0.144			
	Proactive aggressive	−0.004	0.973			
PEP	Grandiose manipulative	0.011	0.921	0.063	0.237	0.062
	Callous-unemotional	−0.131	0.211			
	Impulsive irresponsible	−0.160	0.189			
	Reactive aggressive	0.232	0.059			
	Proactive aggressive	−0.071	0.565			
SCL	Grandiose manipulative	0.040	0.722	0.066	0.204	0.073
	Callous-unemotional	0.006	0.950			
	Impulsive irresponsible	0.225	0.063			
	Reactive aggressive	0.100	0.410			
	Proactive aggressive	−0.230	0.062			

Similarly, within the multi-problem group, we performed four linear regression analyses with HR, RSA, PEP, and SCL change as outcome measures. In each model, the psychopathy and aggression subscales were entered as predictors. None of these models were significant (all *p*s >0.05; see Table 4 for an overview). Bayesian analyses indicate substantial evidence in favor of the null hypotheses with Bayes factors (*BF*_10_; model compared to null model) ranging from 0.03 to 0.04.

**TABLE 4:** ANS reactivity regression models within multi-problem young adults (*N* = 112).

Outcome	Predictor	β	*p*-value β	*R*^2^	*p*-value *R*^2^	*BF*_10_
HR change	Grandiose manipulative	0.163	0.162	0.050	0.394	0.040
	Callous-unemotional	0.098	0.368			
	Impulsive irresponsible	−0.062	0.612			
	Reactive aggressive	0.040	0.742			
	Proactive aggressive	−0.212	0.089			
RSA change	Grandiose manipulative	0.101	0.387	0.059	0.303	0.028
	Callous-unemotional	0.143	0.189			
	Impulsive irresponsible	0.010	0.937			
	Reactive aggressive	−0.094	0.443			
	Proactive aggressive	−0.175	0.163			
PEP change	Grandiose manipulative	−0.071	0.540	0.075	0.170	0.038
	Callous-unemotional	−0.025	0.817			
	Impulsive irresponsible	0.017	0.887			
	Reactive aggressive	−0.009	0.939			
	Proactive aggressive	−0.232	0.061			
SCL change	Grandiose manipulative	−0.086	0.451	0.057	0.301	0.032
	Callous-unemotional	0.119	0.268			
	Impulsive irresponsible	−0.177	0.146			
	Reactive aggressive	−0.114	0.348			
	Proactive aggressive	0.169	0.169			

### Exploratory Comparison Between Multi-Problem Young Adults and Controls

Multi-problem young adults did not differ from controls on any of the psychopathy and aggression scales (all other *p*s >0.05), except for the Reactive aggression scale (*p* = 0.05) on which multi-problem young adults have higher scores than controls. Multi-problem young adults use more cannabis and have a longer history of regular cannabis use than controls (both *p*s <0.001). The average IQ in the multi-problem group was 82.64 (see Table 1 for an overview).

We found no significant differences between the multi-problem group and the control group on the four physiological measurements of the ANS during the 5 min baseline video (all *p*s >0.05; see Table 1 for an overview).

Multi-problem young adults did not differ from the control group in their assessments of the emotion of the boys depicted in the film clips nor did they differ in the emotions they experienced themselves (for both clips and all emotions all *p*s >0.05). The multi-problem young adults and controls did not differ in their autonomic reactions to the film clips (all *p*s >0.05; see Table 5 for an overview of the difference scores for each group). Bayesian analyses indicate anecdotal to substantial evidence in favor of the null hypotheses with Bayes factors (BF10; model compared to null model) ranging from 0.24 to 0.42.

**TABLE 5:** Evaluation of emotions and autonomic responses to sadness.

	Multi-problem young adults (*N* (112)		Healthy controls (*N* = 22)			
		
	*M*	*SD*	*M*	*SD*	*p*	*BF*_10_
**Emotion evaluation (protagonist)**						
Happiness—Mohammed	1.32	0.93	1.00	0.00	0.067	*
Happiness—Champ	1.37	1.11	1.00	0.00	0.106	*
Anger—Mohammed	2.27	1.19	2.00	0.93	0.414	0.375
Anger—Champ	2.26	1.35	2.09	1.19	0.691	0.276
Fear—Mohammed	2.10	1.13	2.41	1.30	0.709	0.421
Fear—Champ	3.32	1.33	3.45	1.14	0.721	0.263
Sadness—Mohammed	4.60	0.84	4.77	0.43	0.609	0.353
Sadness—Champ	4.88	0.35	5.00	0.00	0.106	*
**Emotion evaluation (self)**						
Happiness—Mohammed	1.81	1.15	1.86	0.83	0.832	0.246
Happiness—Champ	1.55	1.05	1.64	1.08	0.740	0.253
Anger—Mohammed	2.05	1.34	2.00	1.11	0.858	0.245
Anger—Champ	1.74	1.22	1.55	1.22	0.505	0.292
Fear—Mohammed	1.35	0.99	1.32	0.78	0.903	0.243
Fear—Champ	1.45	1.06	1.36	0.95	0.709	0.256
Sadness—Mohammed	2.10	1.32	2.45	1.54	0.265	0.412
Sadness—Champ	2.45	1.33	2.55	1.37	0.771	0.250
**Autonomic response (difference from baseline)**						
HR (bpm)—Mohammed	−1.46	4.97	−2.34	3.14	0.426	0.318
HR (bpm)—Champ	−2.66	3.95	−2.50	3.69	0.863	0.244
RSA—Mohammed	−14.22	47.12	−2.15	51.93	0.322	0.397
RSA—Champ	−16.57	34.43	−10.60	26.89	0.445	0.311
PEP (ms)—Mohammed	2.71	16.33	2.22	10.24	0.896	0.252
PEP (ms)—Champ	0.87	13.89	0.18	10.42	0.833	0.255
SCL (μS)—Mohammed	−0.45	0.82	−0.32	0.44	0.298	0.301
SCL (μS)—Champ	−0.29	0.73	−0.17	0.29	0.449	0.309

Emotions were evaluated on a 5-point Likert scale. *BF_10_ could not be computed due to variance of 0 in the control group.

## Discussion

In this study we investigated the relationship between psychopathic traits, aggression, and both the physiological activity and responsivity of the ANS in a large, naturalistic sample of multi-problem young adults. We studied four measures of the ANS (heart rate, respiratory sinus arrhythmia, pre-ejection period, and skin conductance) to capture both SNS and PNS activity. We adopted a dimensional approach as to include the full range of psychopathy and aggression and optimize power.

Contrary to our expectations, within the multi-problem group we found no associations between any of the psychophysiological measurements and their psychopathic traits and aggressive behavior. This is at odds with previous studies(e.g., Latvala et al., 2015; Murray et al., 2016), although other recent studies also failed to find significant associations (Oldenhof et al., 2018; Prätzlich et al., 2018). Additionally, specifically the relationship between low resting heart rate and antisocial behavior has been weakening over the years of research (Portnoy and Farrington, 2015). This may be explained by the “proteus phenomenon,” which entails the effect that in the early phase of investigation, both positive and negative significant findings are published. Later, findings refuting the original results become more attractive and null-findings are published more often. Our null-findings cannot be attributed to problems with the experiment, as autonomic changes from baseline measurements to film clip measurements were present in the right direction (Kreibig, 2010) and clearly visible in both multi-problem young adults and controls, indicating that the task worked appropriately and our autonomic measures are valid. Possibly, the relationship between ANS and antisocial behavior is stronger in childhood and adolescence and diminishes into adulthood. For baseline findings this is a realistic possibility, as meta-analytic effect sizes for adults have shown to be about twice as small as effect sizes in children and adolescents (Portnoy and Farrington, 2015). Another possibility is that because due to its naturalistic nature our sample is necessarily heterogeneous, which could account for diminished effect sizes. This is important as when we try to translate research findings into practical and clinical implications we unavoidably move from more controlled and selected populations and environments to more natural populations and environments. Of course, another explanation for these null-findings is that simply no relation between ANS (re)activity and psychopathy and aggression exists in multi-problem young adults. Finally, we cannot exclude the possibility that autonomic functioning may be hampered specifically in subgroups of individuals with more extreme levels of antisocial behavior, which may be underrepresented in our sample. Although multi-problem young adults present with clear antisocial behavior, they show robust and normative autonomic responses to sadness as measured with HR, RSA, and SCL.

In exploratory analyses, we found no differences between multi-problem young adults and controls on both baseline and responsivity measures of the ANS. Likewise, the groups did not differ in their evaluation of the emotions of the protagonists of the movies nor of themselves, which is at odds with findings from the literature (e.g., de Wied et al., 2012; Maffei et al., 2020). These results are somewhat harder to interpret as multi-problem young adults suffer from a plethora of antisocial problems, but our sample does not differ from controls in terms of psychopathic traits and only slightly on aggression. Given the forensic nature of our sample it seems unexpected that multi-problem young adults would have similar psychopathy scores to community controls. However, more studies have found this to be the case in forensic samples (e.g., Boonmann et al., 2015; Aghajani et al., 2017). It may be representative of the fact that the YPI, contrary to for example the Psychopathy Checklist Youth Version, does not include criminal behavior *per se* in its definition of psychopathy. Additionally, as our control group scores fairly high on measures of aggression compared to other non-offender groups (see Cima et al., 2013) and psychopathy and aggression are positively related, it may be coincidental that we have recruited a control group scoring relatively high on both psychopathy and aggression.

This study is limited by the use of a fairly small control group, because its main purpose was to assess task validity. This provides limited interpretability of the analyses comparing participants and controls, although other studies have used experimental groups of similar size (e.g., de Wied et al., 2009; de Wied et al., 2012). In contrast, our study is strengthened by its large sample size in terms of autonomic responsivity. Whereas studies investigating baseline autonomic activity are more often large and well-powered (e.g., Latvala et al., 2015; Prätzlich et al., 2018), studies looking into autonomic responsivity commonly have group designs with group sizes of 15–35 participants (e.g., Beauchaine et al., 2007; de Wied et al., 2012; Marsh et al., 2008), limiting the generalizability of results.

## Conclusion

In a large, naturalistic sample of multi-problem young adults we found no evidence for a relationship between resting ANS functioning and psychopathy and aggression, nor did we find evidence for a relationship between ANS reactivity and psychopathy and aggression. Our null-findings highlight the need for research with more naturalistic samples, as results may be at odds with findings from clinical samples.

## Data Availability Statement

The raw data supporting the conclusions of this article will be made available by the authors, without undue reservation, to any qualified researcher.

## Ethics Statement

The studies involving human participants were reviewed and approved by the Medical Ethical Committee of the VU University Medical Center. The patients/participants provided their written informed consent to participate in this study.

## Author Contributions

JZ, RM, and AP designed the study. JZ, LD, and M-JL performed data collection. JZ analyzed the data and wrote the first draft JZ and RM interpreted results. All authors contributed significantly to the further writing and finalization of the manuscript, read and approved the final manuscript.

## Conflict of Interest

The authors declare that the research was conducted in the absence of any commercial or financial relationships that could be construed as a potential conflict of interest.

## Footnotes

1 http://www.vu-ams.nl/support/instruction-manual/
